# Crop Mapping Based on Sentinel-2 Images Using Semantic Segmentation Model of Attention Mechanism

**DOI:** 10.3390/s23157008

**Published:** 2023-08-07

**Authors:** Meixiang Gao, Tingyu Lu, Lei Wang

**Affiliations:** 1 Department of Geography and Spatial Information Techniques, Ningbo University, Ningbo 315211, China; gaomeixiang@nbu.edu.cn; 2 College of Geography and Environmental Sciences, Hainan Normal University, Haikou 571158, China; 3 Heilongjiang Province Key Laboratory of Geographical Environment Monitoring and Spatial Information Service in Cold Regions, Harbin Normal University, Harbin 150025, China; wanglei@hljit.edu.cn

**Keywords:** Sentinel-2 images, crop mapping, deep learning, attention mechanism, semantic segmentation

## Abstract

Using remote sensing images to identify crop plots and estimate crop planting area is an important part of agricultural remote sensing monitoring. High-resolution remote sensing images can provide rich information regarding texture, tone, shape, and spectrum of ground objects. With the advancement of sensor and information technologies, it is now possible to categorize crops with pinpoint accuracy. This study defines crop mapping as a semantic segmentation problem; therefore, a deep learning method is proposed to identify the distribution of corn and soybean using the differences in the spatial and spectral features of crops. The study area is located in the southwest of the Great Lakes in the United States, where corn and soybean cultivation is concentrated. The proposed attention mechanism deep learning model, A_2_SegNet, was trained and evaluated using three years of Sentinel-2 data, collected between 2019 and 2021. The experimental results show that this method is able to fully extract the spatial and spectral characteristics of crops, and its classification effect is significantly better than that of the baseline method, and it has better classification performance than other deep learning models. We cross verified the trained model on the test sets of different years through transfer learning in both spatiotemporal and spatial dimensions. Proving the effectiveness of the attention mechanism in the process of knowledge transfer, A_2_SegNet showed better adaptability.

## 1. Introduction

Remote sensing is an emerging technology developed in the early 1960s. Following decades of rapid development, it is widely used in natural environment surveyance, hydrology, meteorology, environmental monitoring, and other fields, and has become a practical and advanced space exploration technology [1,2,3,4]. In the field of agriculture, accurate information regarding crop planting structure is important reference data for the optimization and adjustment of planting structure and the issuing of agricultural subsidies [5,6,7]. The traditional method of manually recording crop type, spatial distribution and other field attributes has the problems of low efficiency and high cost, especially in the face of large-scale near-real-time crop-type monitoring. This is an almost impossible task; therefore, more effective and practical means are needed. In recent years, with the rapid development of aerospace technology, sensor technology, and digital image processing technology, land cover classification based on remote sensing images with high temporal resolution and high spatial resolution using machine learning algorithms has become a very active research area in the application of remote sensing technology [8,9,10]. Multi-spectral data, temporal NDVI data and temporal synthetic aperture radar data, represented by Sentinel and Landsat, have been widely applied to crop classification tasks [11,12,13].

Training a supervised learning model requires a large number of reliable ground reference materials. In the actual operation process, the collection of large-scale crop type labels is largely reliant on field surveys and censuses, supplemented by indoor work to reach completion, which is expensive and time consuming. The scarcity of crop type labels is one of the major challenges in producing high-precision crop classification maps [14]. Using publicly available crop type label datasets is one solution; however, it is easy to encounter the problems of crop type label richness and spatial range, as well as generalization errors and false correlations caused by the use of remote sensing images with different acquisition times and illumination conditions [15]. Another method is to generate virtual labels through semi-supervised learning algorithms such as LabelSpreading. However, problems such as category imbalance and label noise can mislead the network, which will then be memorized by network learning [16]. A typical plot-level crop type map is the Cropland Data Layer (CDL), published by the U.S. Department of Agriculture’s (USDA’s) National Agricultural Statistics Service (NASS). This dataset uses medium-resolution satellite images, farmer report data, and other auxiliary data to generate a national crop type map with a resolution of 30 m [17,18]. This high-precision crop classification map can be used for crop planting area estimation, yield prediction, disaster monitoring, and other applications [19,20].

Most of the early methods used for the classification of remote sensing images are based on statistical mathematical methods, such as maximum likelihood, minimum distance, and Mahalanobis minimum distance [21,22,23]. These methods are linear models based on distance measurement, limited by computing power and dataset size, and only applicable to shallow reasoning. Based on probability statistics, machine learning algorithms are able to obtain hidden, effective, and understandable knowledge from data through inference and sample learning, and to then model the data [24,25]. Traditional machine learning algorithms such as decision tree, random forest, and support vector machine have been used widely in remote sensing image classification. Deep learning is a new research field of machine learning. Different from machine learning, the model designs features by itself and combines low-level features with abstract high-level features to discover distributed feature representations of data. Convolutional neural network, as an important deep learning algorithm, has been widely used in the field of computer vision, such as in target detection, image classification, and image segmentation. Semantic segmentation is a computer vision task involving pixel-level classification, which combines image classification, object detection, and image segmentation, and assigns each pixel to a certain category according to certain rules. Convolutional neural networks are favored by many researchers because of their powerful feature extraction ability and good segmentation effect. It has become the most used algorithm in semantic segmentation tasks [26,27]. Although convolutional neural network is a powerful model and has achieved great success in computer vision tasks, continuous convolution will cause changes in the number of feature maps. Pooling operations will also affect the extraction of a pixel’s spatial features in the process of upsampling and downsampling, resulting in the loss of image context information, affecting the performance of the model. For this reason, the attention mechanism was introduced into computer vision, the principle of which is to imitate the human cognitive process of selectively focusing on important information and ignoring secondary information [28]. The attention mechanism has achieved success in tasks such as semantic segmentation [29], image classification [30], and object detection [31].

Among various semantic segmentation networks, SegNet [32] has been proposed for solving image semantic segmentation tasks related to autonomous driving and intelligent robots, and has also been widely used in recent years in the field of remote sensing image classification [33]. Sentinel-2 can cover 13 spectral bands with a spatial resolution of 10 m and contains a wealth of spectral and spatial features. However, in many studies, classification has been performed more on the basis of the spectral features of ground objects, while spatial features have not been effectively utilized. In this study, a model, A_2_SegNet, is proposed for the identification of crop distribution. First, SegNet is improved by introducing an attention mechanism to simultaneously enhance the network’s spatial and spectral feature extraction capabilities. The normalized vegetation index (NDVI) and the red-edge Normalized Vegetation Index (NDVI_705_) generated by single-phase Sentinel-2 images are used to design eight features, including six original bands. Relevant studies have shown that the combination of vegetation index and other bands has a better effect on extracting vegetation information than the combination of original bands [34]. The input image is segmented using A_2_SegNet to generate the crop distribution map.

## 2. Study Area and Experimental Data

### 2.1. Study Area

**Figure 1:**
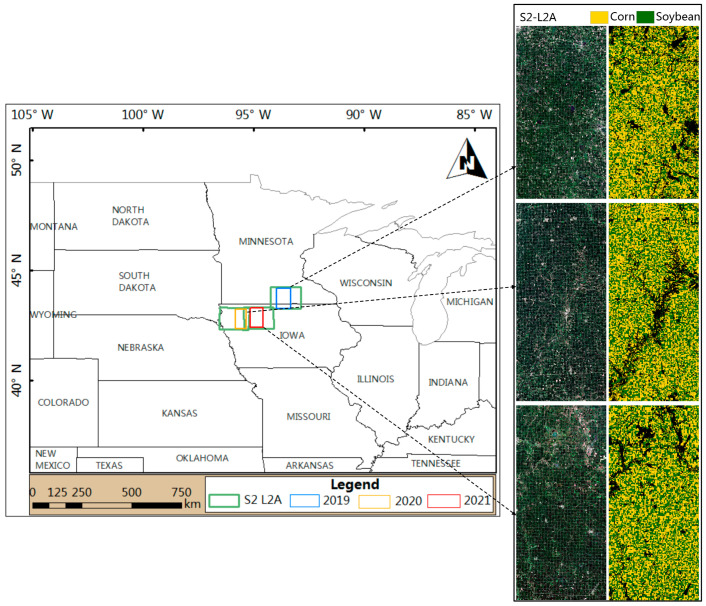
Location of the study area. The green box represents the coverage of the three Sentinel-2 images, while the blue box, yellow box, and red box represent the research area in 2019, 2020, and 2021, respectively. The resolution of the crop land cover data is 30 m.

The study area (96°1′35″ W to 93°14′18″ W, 42°19′23″ N to 44°8′32″ N) is located in the southwestern Great Lakes states of Iowa and Minnesota in the United States, which are major producing areas of corn and soybean. It has a temperate continental climate. There is a winter and a summer. The average temperature in January in the northwest is −9 °C, while it is −4 °C in the southeast. The average daytime temperature in July is 34 °C, the average annual precipitation in the northwest is 711 mm, while it is 864 mm in the south. The location of the research area is shown in Figure 1. The study area was about 5145.6, 4458.3 and 4067.2 square kilometers in 2019, 2020 and 2021, respectively. Table 1 lists the cultivated land data layer (CDL, https://croplandcros.scinet.usda.gov/ (accessed on 23 April 2023)), including the specific sample size of corn and soybeans for different years in the study area.

### 2.2. Sentinel-2 Datasets

**Figure 2:**
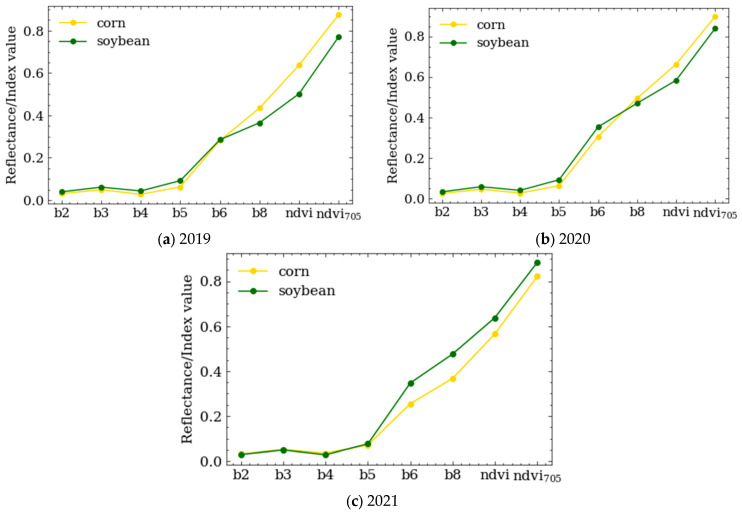
Average spectral curves of corn and soybeans in different years after data reconstruction.

**Figure 3:**
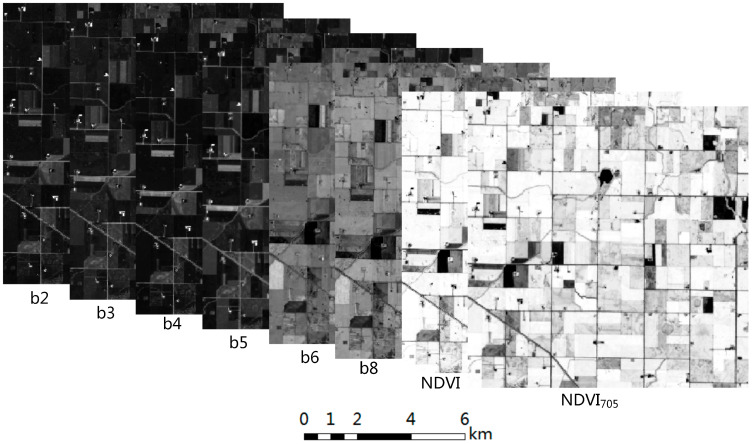
Composition of the multi-channel image, all bands are resampled to a resolution of 30 m.

The images of the three views Sentinel-2 were downloaded from ESA’s official website (https://scihub.copernicus.eu/dhus/#/home (accessed on 23 April 2023)), the data were fine after performing the correction and geometric correction for the apparent atmospheric reflectance L1C products. In order to make full use of the multi-dimensional information in the multispectral images and in consideration of the important role played by vegetation index in crop classification, eight features (band 2, band 3, band 4, band 5, band 6, band 8, NDVI, and NDVI_705_) were designed in this study. The processing process for the Sentinel-2 data was as follows: (1) Sen2Cor software was used to perform atmospheric correction and generate L2A products; (2) the data of 6 bands were resampled to 30 m resolution using the batch processing program by IDL; (3) NDVI and NDVI_705_ were generated using the Raster Calculator on the ArcGIS platform (subsequent processing was carried out using this platform); (4) the Composite Bands tool was used to complete band synthesis; (5) the Clip tool was used to cut out the research area. The spectral curves of crops in different years are shown in Figure 2. The final data generated are 8-channel images, and the grayscale images of each channel are shown in Figure 3. The wavelength and spatial resolution of the 8 features are shown in Table 2.

### 2.3. Ground Truth

**Figure 4:**
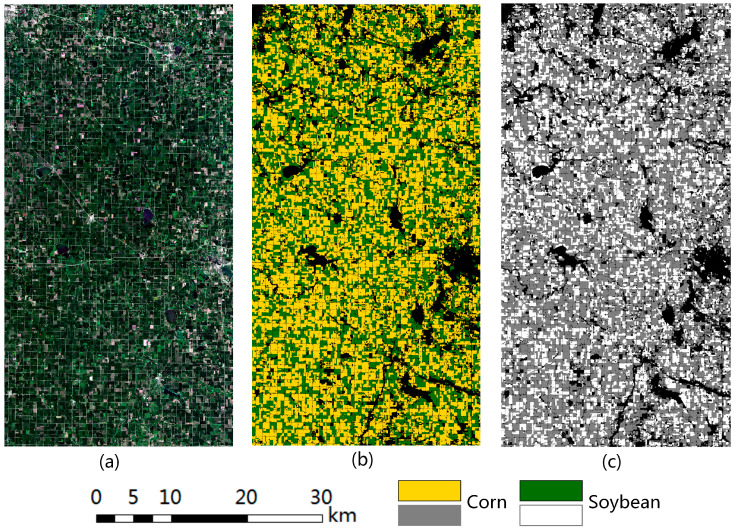
The study area in 2019. (**a**) Sentinel-2 true color image. (**b**) Ground truth of the CDL. (**c**) Extracted crop type label, where the data are stretched to 0−255.

The USDA’s CDL provides spatial information on crop acreage and has become a popular data source for characterizing changes in cultivated land in the United States over the past decade [35]. Since 2008, CDL has covered 48 states and released 134 land cover categories based on satellite images and ground data every year, making it the largest public crop classification dataset in the world. In recent years, some scholars have compared CDL with other datasets and proved its reliability [36]. Therefore, CDL datasets corresponding to the Sentinel-2 datasets in 2019, 2020, and 2021 were selected as the reference data, and two crops of corn and soybean were extracted to generate ground object category labels. The true color images and the CDL reference data of the study area in 2019 are shown in Figure 4.

### 2.4. Training and Test Sample

**Figure 5:**
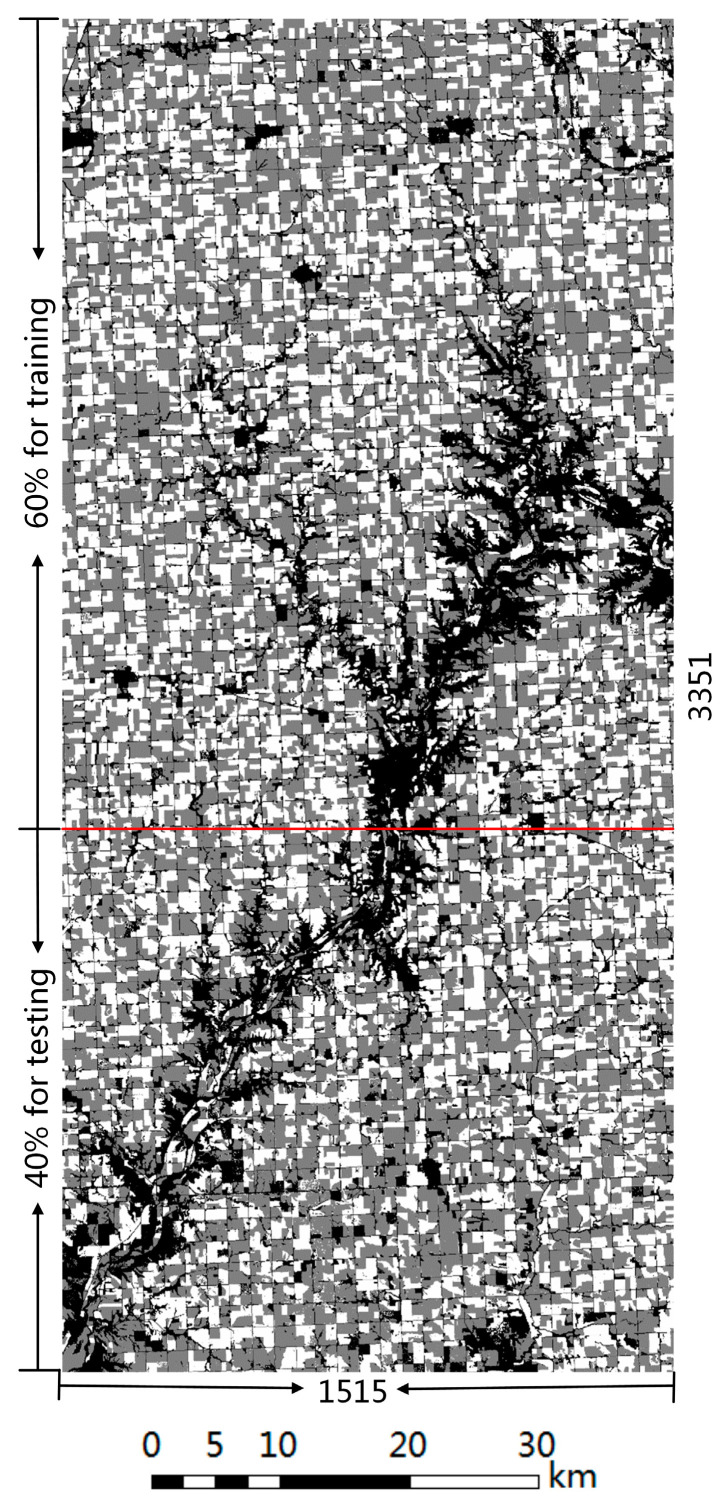
Construction of sample images from the extracted crop type label, where the label data is stretched to 0–255. The red line is the dividing line between the training set and the test.

**Figure 6:**
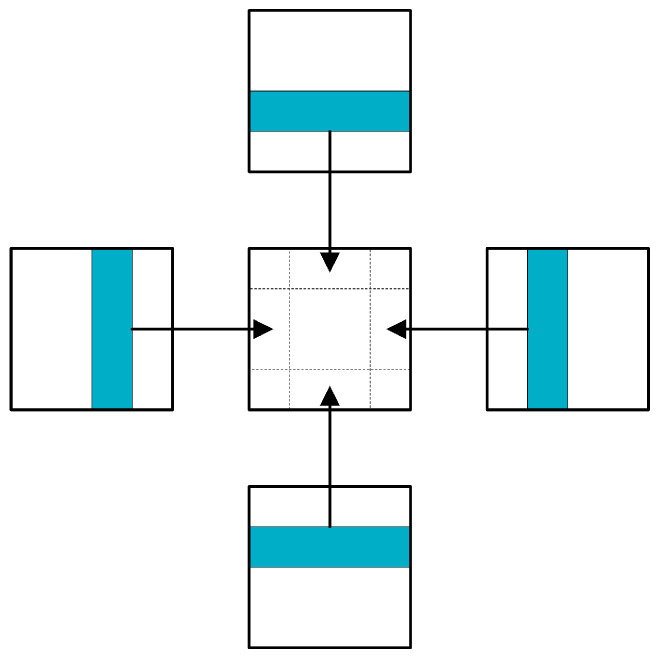
Strategy of ignoring edge prediction results.

The data used for model training and testing are 8-channel images (see Figure 3). Sentinel-2 data of different years are divided into training sets and test sets according to the ratio of 6:4. Taking 2020 data as an example, Figure 5 illustrates the partitioning rules. Due to the limitation of GPU computing power, the size of the image input to the model should not be too large. In this study, the size of the input image is limited to 256 × 256 × *C*, where *C* is the number of image channels. The training set area of each Sentinel-2 image and the crop category label image were randomly cropped into 800 small images with a size of 256 × 256 × 8, of which 600 images were used for model training and 200 images were used for model validation. The test set is used to evaluate the generalization ability of the final model, and the image size in the test set is consistent with that in the training set. When the model is used for prediction, the prediction accuracy is low, due to the edge region of the image offering insufficient contextual information. Therefore, the method of ignoring the edge region is adopted when the image of the test set is generated. The edge regions in the four directions of the image are replaced with the middle regions of adjacent images (see Figure 6). Similarly, the same processing method is also employed when splicing the prediction results.

## 3. Crop Mapping Based on Deep Learning

### 3.1. Attention Mechanism in Artificial Neural Network

When processing large volumes of information, the human visual nervous system naturally and effectively captures areas with prominent information in complex scenes, and key information will receive special attention. Inspired by biological visual cognitive mechanisms, artificial neural networks introduce the attention mechanism into computer vision tasks. The essence of the attention mechanism is to extract the most prominent attributes of the input scene data. The dynamic allocation of image feature weights achieves the purpose of improving network performance [37]. Since the first Recurrent Attention Model (RAM) based on the attention mechanism was proposed [38], the attention mechanism has played an increasingly important role in computer vision, natural language processing, and other fields. Channel attention, spatial attention, temporal attention, and branch attention networks have been proposed based on the data domain.

Channel attention pays extra attention to goal-related semantic information in the channel dimension, such as SENet attention networks, which model channel information and adaptively re-correct the strength of the feature responses between channels through the global loss function of the network. FcaNet first proved that global average pooling was a special case of discrete cosine transform, and then, on this basis, proposed a new multi-spectral channel attention, creatively bisecting feature graphs, applying 2D discrete cosine transform, and generating the weights of each channel after vector splicing through the fully connected layer and the activation function [39]. Spatial attention processes the most important region in the image and retains key information during the spatial transformation of the image. For example, the Sampling Equivariant Self-Attention Network processes the most important region in the image and retains key information during the spatial transformation of the image [40]. Temporal attention can be seen as a dynamic selection mechanism in which time nodes should be focused on in temporal data, and is commonly used in video processing. In branch attention networks, convolution kernels of different sizes form networks with multiple branches, and multi-scale convolution kernels generate perceptive fields of different view fields. Large convolution kernels can improve model performance by obtaining global features, while small convolution kernels reduce computational load and enhance the model’s nonlinear representation [41,42]. The attention mechanism can be expressed as follows:

(1) $$Attention=f\left(g\left(x\right),x\right)$$

where *g*(*x*) represents the process of generating attention, and *f*(*g*(*x*), *x*) represents the process of processing input feature x in combination with attention *g*(*x*). The attention mechanism was initially applied in natural language processing tasks. In recent years, many scholars have carried out a large number of studies in the field of combining deep learning and visual attention mechanisms, and have introduced a large number of channel attention models and spatial attention models. At the same time, mixed channel and spatial attention models have also become a research hotspot. The lightweight attention module CBAM adds attention to the channel domain and the spatial domain by combining maximum pooling and mean pooling, which has the advantages of fewer parameters and low computational overhead [43]. The RGA abandons the traditional method of learning attention through local convolution; instead, it mines valuable clustering knowledge in the global structure, and then builds attention through learning functions. The proposed global attention module can be applied in both channel and space dimensions, and the effectiveness of this model has been verified in pedestrian re-recognition tasks [44]. Coordinate Attention is a spatial-information-imbued attention mechanism designed for mobile neural networks. It innovatively incorporates spatial information into channels and generates coordinate attention by encoding channel relationships and long-term dependency relationships. Its advantage is that it can obtain cross-channel information that is sensitive to spatial position [45].

### 3.2. Attention Mechanism Neural Network A_2_SegNet

**Figure 7:**
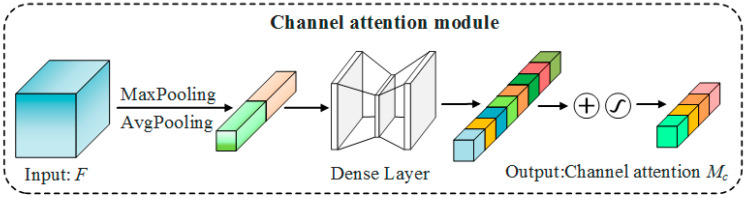
Channel attention: the module learns features in the channel dimension, different colors indicate a single channel or a combination of channels.

In this study, SegNet is improved by inserting a CBAM module to add attention to the channel dimension and the spatial dimension, and the A_2_SegNet semantic segmentation network is proposed. The structure of the channel attention module is shown in Figure 7.

Channel attention is modeled for feature channels. Important features are promoted and unimportant features are suppressed according to the importance of the channels. The process for the generation of channel attention is as follows: The first step involves applying global maximum pooling and global average pooling to an input feature F with dimensions *W* × *H* × *C*. This results in two 1 × 1 × *C* feature vectors, *F_avg_* and *F_max_*. Then, the two vectors are respectively input into a neural network composed of two fully connected layers. After passing through the first fully connected layer, the dimensions of *F_avg_* and *F_max_* are reduced to 1 × 1 × *C*/*r*, where r is the compression ratio. After the second fully connected layer, *F*_avg_ and *F*_max_ return to 1 × 1 × *C*, and finally, the output vectors are summed to generate channel attention. The calculation process is as follows:

(2) $$M_{c}\left(F\right)=\sigma \left(MLP\left(AvgPool\left(F\right)\right)+MLP\left(MaxPool\left(F\right)\right)\right)$$

(3) $$=\sigma \left(W_{1}\left(W_{0}\left(F_{avg}\right)\right)+W_{1}\left(W_{0}\left(F_{max}\right)\right)\right)$$

In the formula, *σ* represents the sigmoid activation function, *F* represents the input feature, and *W*_0_ and *W*_1_ are the weights of the two fully connected layers.

**Figure 8:**
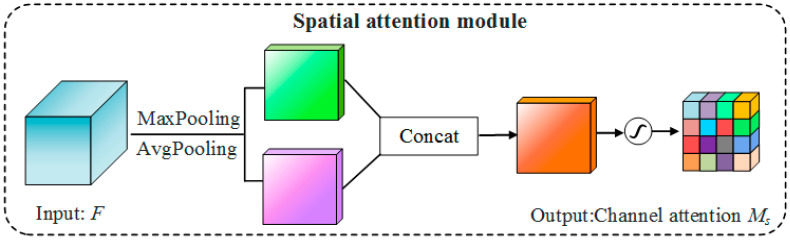
Spatial attention: the module captures important regional features.

The process for the generation of spatial attention is as follows: First, two *W* × *H* × 1 dimension feature vectors are obtained by applying maximum pooling and average pooling along the channel axis of input feature *F*; then, the two features are spliced, and convolution operations are performed; finally, spatial attention is generated by the sigmoid activation function, the structure of which is shown in Figure 8.

The calculation process is as follows:

(4) $$M_{s}\left(F\right)=\sigma \left(f^{7\times 7}\left(\left[AvgPool\left(F\right);\;MaxPool\left(F\right)\right]\right)\right)$$

(5) $$=\sigma \left(f^{7\times 7}\left(\left[F_{avg};F_{max}\right]\right)\right)$$

In the formula, σ represents the sigmoid activation function, *F* represents the input feature, and *f*^7×7^ represents the 7 × 7 convolution kernel.

**Figure 9:**
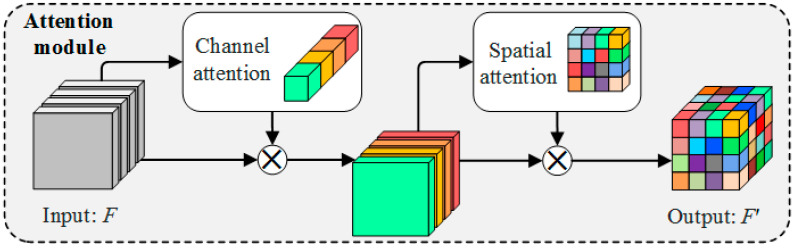
Architecture of the CBAM attention module: channel attention and spatial attention are combined through concatenation.

Channel attention and spatial attention complement each other and can be inserted into the neural network in combination. Since the attention module does not change the size of the output feature map, they can be placed anywhere in the network. Figure 9 illustrates the structure of CBAM, where the channel attention module is placed in front and the spatial attention module behind.

The attention module has parameters; therefore, adding it will increase the complexity of the model, and can improve its performance in the underfitting state. For the model in the overfitting state, adding parameters may aggravate the overfitting problem, and the performance may remain unchanged or even decline.

**Figure 10:**
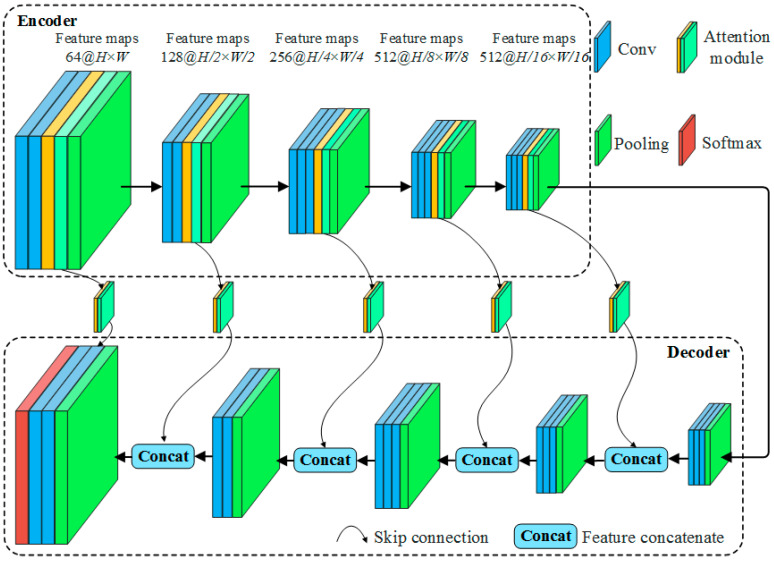
An illustration of the A_2_SegNet architecture; the network is composed of an encoder and a decoder.

Structurally, CBAM consists of both spatial and a channel attention modules, both of which use global average pooling to extract abstract features and generate attention weights, and then establish the relationship between these weights and attach them to the original space or channel features. From a location perspective, CBAM should not be positioned behind the input layer of the network. This is because the spatial feature map is excessively large, while the number of channels is relatively small. Additionally, the weights of the extracted channels are too generalized to be able to effectively converge on specific features. The extracted spatial attention is sensitive and difficult to learn due to the small number of channels and the lack of generality, and is more likely to cause negative effects. Similarly, CBAM should not be placed in front of the output layer of the network. In A_2_SegNet, we inserted five CBAMs in the encoder part, while the original structure was preserved unchanged in the decoder part. At the same time, we abandoned the original SegNet network record maximum position index strategy, and replaced it with a skip connection, which also included CBAM. The effectiveness of skip connections when fusing the coarse features of different scales extracted by encoders with high-level semantics has been demonstrated in many neural networks [46,47,48,49]. The network structure of A_2_SegNet is shown in Figure 10.

The CBAM module does not change the dimension of the input features and can be placed behind any convolution layer. However, the large number of fully connected layer parameters in CBAM increases the computational overhead. Therefore, it is impractical to insert CBAM modules behind each convolution layer. The five CBAM modules of A_2_SegNet are located in the middle of the convolution layer and the pooling layer of each stage of the encoder. Within the CBAM, modules are arranged in the order of channel attention module followed by spatial attention module. The A_2_SegNet encoder consists of five stages. The first stage is composed of two convolution layers, one CBAM and one maximum pooling layer. The convolution kernel size of the convolution layer is uniformly set to 3 × 3, with 64 feature maps. Taking the input layer’s image size as (256, 256, *C*) for illustration, the first downsampling operation yields output feature maps with a size of 128 × 128 × 64. In the second stage, two convolutions (also with a 3 × 3 kernel size) and one CBAM generate 128 new feature maps. Subsequently, the second downsampling operation reduces the output dimensions to 64 × 64 × 128. The third stage is composed of three convolution layers, one attention module and one pooling layer. The output feature maps are 32 × 32 × 256. In the fourth and fifth stages, the input images are further compressed, and the number of features is increased to 512. Attention modules are added in both stages, and the feature dimension of the final output from the encoder to the decoder is 8 × 8 × 512. In the decoder part, after the first upsampling and three convolutions, 16 × 16 × 512 the output feature map is concatenated with the output feature map of the attention module in the fifth stage of the encoder to form a new feature map with dimensions of 16 × 16 × 1024, and so on. After the second through fourth feature map concatenations, the feature maps are 32 × 32 × 768, 64 × 64 × 384, and 128 × 128 × 192, respectively. The feature maps of the output of the fifth skip connection have dimensions of 256 × 256 × 64. After feature map concatenation with the results of the fifth upsampling from the decoder (feature maps with dimensions of 256 × 256 × 192), the output has dimensions of 256 × 256 × 256. Finally, after two convolution operations, the activation function in SoftMax outputs the classification result.

### 3.3. Model Construction and Training Strategy

In this study, A_2_SegNet was implemented using the deep learning framework Keras (with Tensorflow as the backend) in the Python language. Model construction and training were carried out using the following hardware and software configurations:

- Operating system: Windows 7 Professional SP1.
- CPU configuration: Intel(R) Xeon(R) CPU E5-1650 v4 3.6 GHz 12-core processor.
- Memory size: 64 GB.
- Graphics configuration: NVIDIA Quadro P4000.
- Programming language: Python.
- Deep learning development frameworks: TensorFlow, Keras.

The goal of deep learning is to learn the inherent law and representation level of sample data. Convolutional neural networks calculate the weight and deviation of neurons through convolutional operations. The output of a convolutional layer (or deconvolutional layer) is a set of feature graphs, in which each feature graph is the result of a convolution operation between fixed-weight parameters in the unit and input data, and finally more advanced features are extracted through the full connection layer. The classifier creates a mapping between the input image and the real label. During model training, in this study, the initial configuration scheme was selected to be fast with minimal resource consumption in order to make hyperparameter adjustment more efficient. In order to avoid shock, the learning rate should not be too large, and is usually set to 0.01. Here, the initial learning rate was set to 0.1, and the learning rate was dynamically adjusted by observing loss. The batch size was set to 8; this was a choice made to ensure the stability of training and the computational efficiency of the hardware. The weight attenuation was set to 0.001, the epoch was set to 150, and the optimization function used Adam. All the training processes adopted the early stopping strategy: in cases where the training accuracy did not increase for 30 consecutive rounds, the training was stopped, and the model with the best performance was saved.

Both SegNet and CBAM are lightweight, and the size of the sample information used was small. This allows A_2_SegNet to fit the entire sample space effectively, avoiding overfitting and underfitting to a large extent. Additionally, the numbers of samples for corn and soybean were the same, eliminating any issues related to category imbalance. Therefore, no data augmentation was used in the training process.

### 3.4. Evaluation Metrics

In the field of image segmentation, evaluation model performance is mainly judged by three indicators: Overall Accuracy (OA), Mean Intersection over Union (MIoU), Kappa coefficient, as defined in Equations (6)–(8). OA quantifies the proportion of correctly classified samples among all samples. Intersection over Union (IoU) is a standard for measuring the accuracy of the detection of the corresponding object in a specific dataset. Since the object detection algorithm generates the post box of the target object, the definition of IoU is the ratio of the intersection and the union of the “predicted border” and the “marked border”. The MIoU performs inference calculation separately for each data category, divides the intersection of the predicted area and the actual area by its union, and then finds the average of the results obtained for all categories. The Kappa coefficient is an index used for consistency testing to measure the effect of classification. The value range is [−1, 1], and is usually greater than 0; the larger the value, the better the performance of the model.

(6) $$OA=\frac{1}{N}\sum_{i=1}^{k}N_{ii}$$

(7) $$MIoU=\frac{1}{k+1}\sum_{i=0}^{k}\frac{N_{ii}}{\sum_{j=0}^{k}N_{ij}+\sum_{j=0}^{k}N_{ji}-N_{ii}}$$

(8) $$Kappa=\frac{N\sum_{i=1}^{k}N_{ii}-\sum_{i=1}^{k}\left(N_{i+}\times N_{+i}\right)}{N^{2}-\sum_{i=1}^{k}\left(N_{i+}\times N_{+i}\right)}$$

where *N* is the total number of samples; *k* is the total number of categories; *i* is the number of classification results; *j* is the number of verification categories; *N_ii_* is the number of samples assigned to the correct category; *N_jj_* is the number of samples assigned to the correct category in the verification sample; *N_i_*_+_ and *N*_+*i*_ are the total numbers of samples in row *I* and column *i*, respectively; and *N*_+*j*_ is the total number of samples classified into class *j* in the actual classification.

## 4. Experimental Results

In this section, we first analyze the feasibility of applying deep learning algorithms to crop classification based on reconstructed Sentinel-2 data, and then cross-validate the generalization ability of the model using test sets and pre-trained networks of different years and examine the performance of the proposed method.

### 4.1. Features Learned Using the Deep Learning Model

A popular nonlinear dimension reduction technique is t-SNE (t-Distributed Stochastic Neighbor Embedding) [50]. It is mainly used to visualize high-dimensional data and to understand and verify data or models. Here, t-SNE was used to analyze the spatial heterogeneity of Sentinel-2 data obtained in different years and the data processed by deep learning model. A total of 3000 samples were randomly selected from corn and soybean, and the original features of these samples and the features extracted from the network were reduced in dimension and projected onto a two-dimensional plane to measure the difference between the two types of feature.

**Figure 11:**
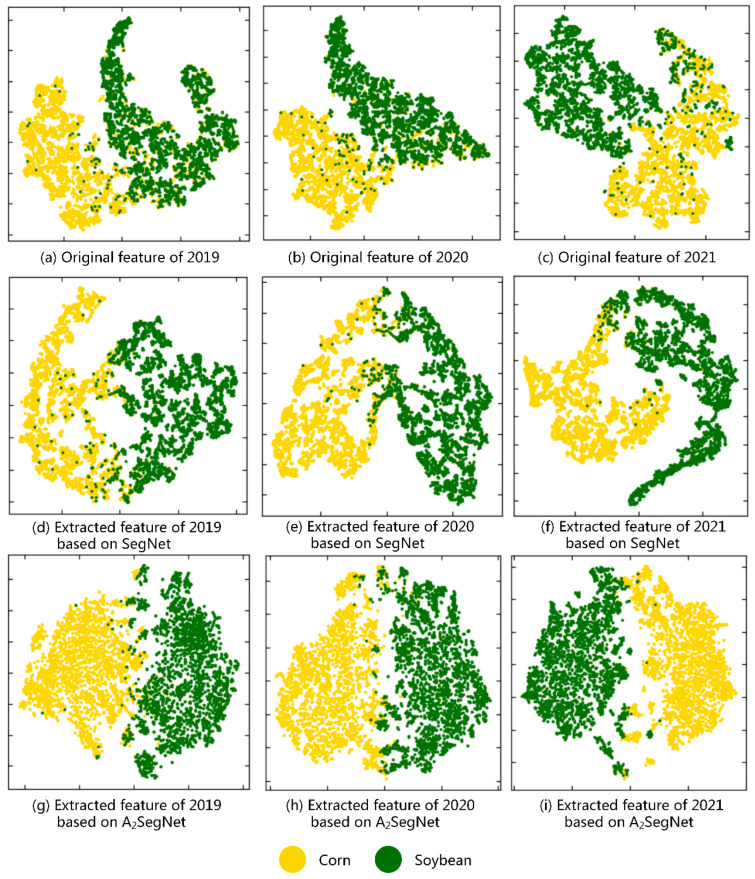
Feature visualization of Sentinel-2 image using t-SNE based on different models for corn and soybean in the years 2019, 2020, and 2021. The axes represent the spatial coordinates of the high-dimensional features projected onto a 2D plane.

As shown in Figure 11, after t-SNE projection onto a two-dimensional plane, the separability of the original features and the features extracted by the deep learning model could be qualitatively analyzed in different categories. The original features of the three different years all showed problems related to large overlaps of samples of different categories and unclear classification boundaries. Based on the features learned from the model, corn and soybean have better classification than the original features. The method proposed in this study generates more discriminative features, the overlap of non-homogeneous samples is greatly reduced, the classification boundary is clear, and the classification of homogeneous sample points is more concentrated and tighter. In summary, features extracted by deep learning models are more separable from each other than the original input features.

### 4.2. Crop Identification Accuracy

The evaluation results of a comparison of the five classification models are shown in Table 3. It can be seen from the OA, MIoU, and kappa that, in general, the performances of deep learning algorithms are better than those of machine learning algorithms. Among the four semantic segmentation networks, A_2_SegNet has the highest evaluation index, followed by UNet++ and UNet, while SegNet has the lowest accuracy. The performance of A_2_SegNet with the introduction of the attention mechanism was significantly improved. Compared with SegNet, taking the 2019 forecast data as an example, OA, MIoU, and kappa increased by 3.46%, 6.64%, and 5.62%, respectively. Similarly, in 2020 and 2021, the performance of A_2_SegNET improved significantly. There are obvious performance differences between the two models.

### 4.3. Crop Prediction Results

**Figure 12:**
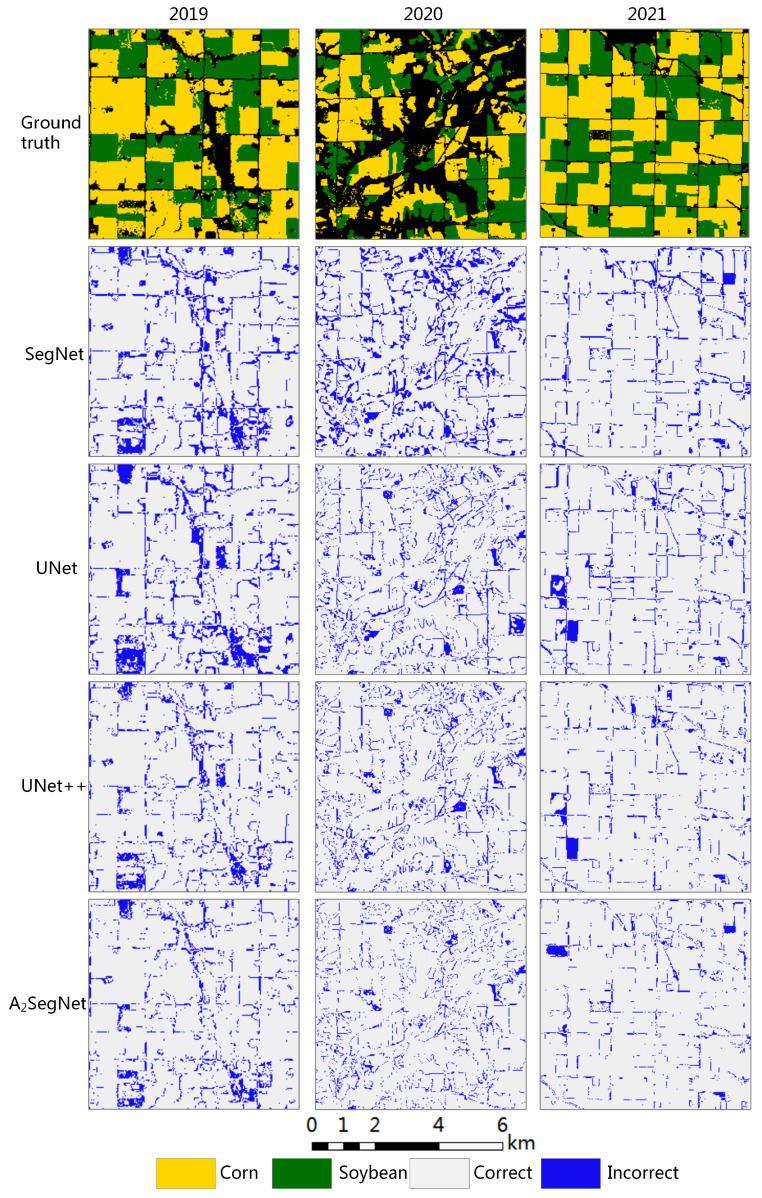
A randomly selected 256-pixel × 256-pixel area, with misclassified samples from different years in the same crop planting areas; blue represents areas of misclassification.

A crop planting area with a size of 256 × 256 was selected to conduct a comparative analysis of the misclassification in different years, and the results are shown in Figure 12. In general, the misclassified regions of all deep learning models are mainly distributed at the junctions of different plots and the fragmentation areas of cultivated land landscapes, while the misclassification of plots with larger regular areas is relatively rare, which is mainly due to the existence of mixed pixels at the boundary affecting the judgment of the model. This situation is particularly evident in the 2020 data in Figure 12. The classification results of UNet and UNet++ are similar, with both being better than SegNet, mainly because UNet and UNet++ fully integrate the low-level features of the downsampling stage with the high-level features of the upsampling stage through the skip connection, thus producing a better classification effect. SegNet is used for fast upsampling of decoders only by recording the maximum pooled location index, which is sparse and leads to a lot of boundary information loss.

By comparing the classification results of A_2_SegNet and SegNet for all years, it is easy to find that the error classification of the former at the block boundary is significantly reduced. This is due to the adaptive feature correction of the input feature map produced using channel attention and spatial attention, and the dynamic optimization of the important information in the original input image. Among these, channel attention learns the important features of the input images by means of maximum pooling and average pooling, and can capture two different pieces of spatial context information with little computational overhead. Spatial attention only pays extra attention to the location of the feature, which makes it easier to learn the input sample, and the spatial information of the plot boundary can be fully utilized. In addition, A_2_SegNet retains the skip connection, and also introduces the dual attention mechanism in the skip connection to ensure that the final output feature map contains more low-level semantics, so that the classification result is more refined.

### 4.4. Transferability Comparison of Deep Learning Models

The degree of adaptability of a deep learning model to new data is an important indicator for evaluating the model’s generalization ability. In order to further test the performance of the attention mechanism neural network proposed by us, the model used here was subsequently migrated in time and space, and the trained model was cross-validated with the test sets of different years (for example, the models generated from the 2019 training data were applied to the 2020 and 2021 test sets, respectively).

**Figure 13:**
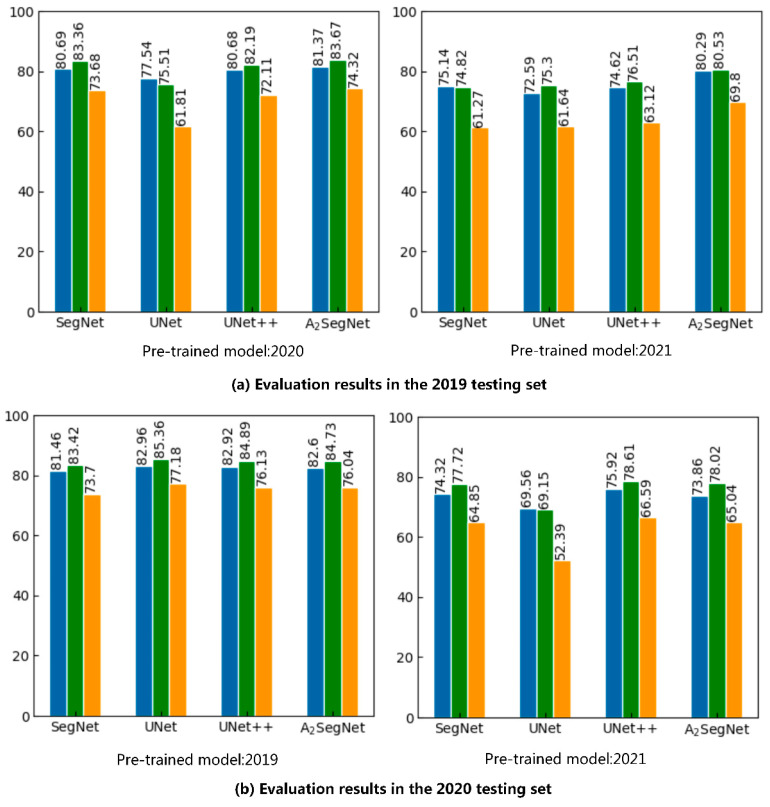

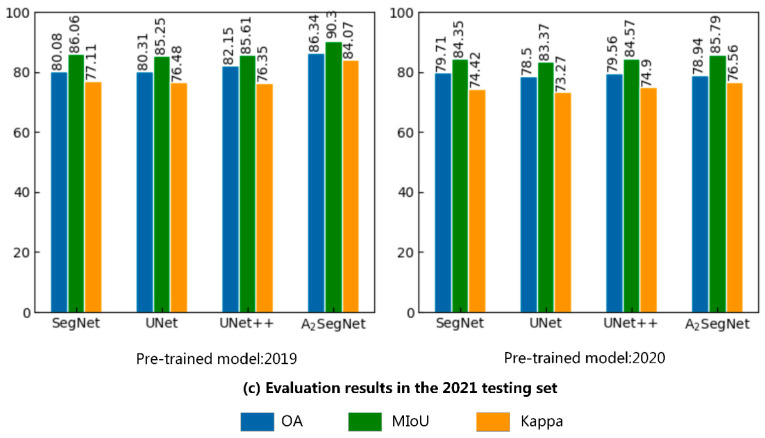
The results of generalization ability test. (**a**) Trained using the 2020 and 2021 training set, tested using the 2019 testing set; (**b**) trains using the 2019 and 2021 training set, tested using the 2020 testing set; (**c**) trained using the 2019 and 2020 training set, tested using the 2021 testing set.

The specific results are shown in Figure 13. It can be seen from the figure that the generalization performances of A_2_SegNet and UNet++ are better than that of SegNet and UNet, and A_2_SegNet, in particular, shows better robustness. Only the 2021 pre-trained model had an A_2_SegNet MIoU of less than 80% when tested on the 2020 data, but even then it reached 78.02%, and the 2019 pre-trained model had an MIoU of 90.3% on the 2021 test set. By comparing the classification results of A_2_SegNet and SegNet, it was found that the generalization performance of A_2_SegNet was better than that of SegNet in all cross-validations, indicating that the attention mechanism can help the model focus more on foreground features and enhance high-level semantics, thus improving the generalization ability of the model. All in all, A_2_SegNet showed stronger adaptability in the process of time and space migration, and can serve as a reference for a wide range of crop mapping applications in the future.

## 5. Conclusions and Discussion

The combination of publicly available remote sensing satellite data with advanced deep learning algorithms can be used to extract a wide range of crop distribution information. Here, we proposed a crop mapping method based on a semantic segmentation network incorporating an attention mechanism to identify maize and soybean by reconstructing Sentinel-2 multispectral data (original band integration normalized vegetation index and red edge normalized vegetation index). In order to make full use of the spectral and spatial features of crops, in this study, a series network of spectral attention and spatial attention was designed to extract the empty spectral features. Hidden feature analysis showed that the attention mechanism model was better able to deal with the complex relationship between the original data and the label of a specific category. This was demonstrated by the effect of sample separability between the original feature and the learned hidden feature. In the deep learning model migration experiment, the proposed A_2_SegNet model demonstrated better adaptability and no obvious performance degradation during time- and space-based migration. The reason for this is that the knowledge transfer focuses on important information, and therefore focuses on the information that is most important to the task in the target domain.

Although deep learning methods have great advantages in terms of accuracy compared to traditional machine learning methods, in practical application they are still restricted by some factors. First, crop sample labeling is time-consuming and laborious work, and is very expensive. Large-scale, high-precision and multi-class datasets like CDL are very rare. Secondly, it is often difficult for optical remote sensing data to obtain a wide range of effective data due to the influence of cloud cover and rainy weather. Finally, the adaptability of the trained model to crop mapping on a global scale is unclear. In future work, we will carry out research in the direction of crop classification based on multi-spectral time series image data and time series synthetic aperture radar data based on deep learning algorithm, in order to further enhance the generalization performance of the model, and apply the deep learning framework proposed in this paper to the supplementary monitoring tool for crop area extraction and agricultural subsidy payment.

## Figures and Tables

**Table 1:** The number of samples of corn and soybeans and the area occupied for the different years.

Year	Crop Types	Sample Count	Area/km^2^
2019	Corn Soybean	2,638,516 1,817,173	2374.7 1635.5
2020	Corn Soybean	2,266,755 1,826,571	2040.1 1643.9
2021	Corn Soybean	2,143,880 1,880,755	1929.5 1692.7

**Table 2:** Features designed in this study, eight original features and two artificial features were applied to the experiment.

Band	Description	Central Wavelength (nm)	Bandwidth (nm)	Spatial Resolution (m)
band 2	Blue	490	98	10
band 3	Green	560	46	10
band 4	Red	665	39	10
band 5	Vegetation Red Edge	705	20	10
band 6	Vegetation Red Edge	740	18	10
band 8	*NIR*	842	133	10
NDVI	$\frac{NIR-R}{NIR+R}$	-	-	10
EVI	$2.5\times \frac{NIR-R}{NIR+6R-7.5B+1}$	-	-	10

**Table 3:** Comparison of the classification accuracy of random forest and four different deep learning models; bold indicates the best classification performance, and the method proposed in the paper achieves the best classification accuracy.

Year	Evaluation Index	RF	SegNet	UNet	UNet++	A_2_SegNet
2019	OA	0.8571	0.8807	0.8879	0.9036	**0.9153**
	MIoU	0.7236	0.7585	0.7765	0.8024	**0.8249**
	Kappa	0.7742	0.8092	0.8234	0.8469	**0.8654**
2020	OA	0.8879	0.8713	0.8991	0.9101	**0.9268**
	MIoU	0.7785	0.7438	0.7954	0.8154	**0.8472**
	Kappa	0.8254	0.7973	0.8421	0.8592	**0.8856**
2021	OA	0.9013	0.9019	0.9188	0.9339	**0.9419**
	MIoU	0.7687	0.7537	0.8026	0.8324	**0.8494**
	Kappa	0.8378	0.8362	0.8662	0.8908	**0.9042**

## Data Availability

Not applicable.
